# Case report: A patient with brachio-cervical inflammatory myopathy was misdiagnosed as flail arm syndrome

**DOI:** 10.3389/fimmu.2024.1378130

**Published:** 2024-07-03

**Authors:** Hui Sun, Xiao-Jing Wei, Ye Han, Yong-Chun Wang, Zi-Yi Wang, Xue-Fan Yu

**Affiliations:** Department of Neurology and Neuroscience Center, The First Affiliated Hospital of Jilin University, Changchun, China

**Keywords:** brachio-cervical inflammatory myopathy, flail arm syndrome, myositis, misdiagnosis, case report

## Abstract

Brachio-cervical inflammatory myopathy (BCIM) is a rare inflammatory myopathy characterized by dysphagia, bilateral upper limb atrophy, limb-girdle muscle weakness, and myositis-specific antibody (MSA) negativity. BCIM has a low incidence and is commonly associated with autoimmune diseases. We present a case report of a 55-year-old man with progressive upper limb weakness and atrophy, diagnosed with flail arm syndrome (FAS). The initial electromyography revealed extensive spontaneous muscle activity and increased duration of motor unit potentials (MUPs). During follow-up, evidence of myogenic damage was observed, as indicated by a decreased duration of MUPs in the right biceps muscle. Laboratory and genetic testing ruled out hereditary or acquired diseases. Negative serological antibodies for myasthenia gravis. Hereditary or acquired diseases were ruled out through laboratory and genetic testing. Whole-body muscle magnetic resonance imaging (MRI) showed extensive edema and fat replacement in the bilateral upper limbs, scapular, and central axis muscles, while the lower extremities were relatively mildly affected. Muscle biopsy revealed numerous foci of inflammatory cells distributed throughout the muscle bundle, with predominant CD20, CD138, and CD68 expression, accompanied by a light infiltration of CD3 and CD4 expression. The muscle weakness improved with the combination of oral prednisone (initially 60 mg/day, tapered) and methotrexate (5 mg/week) treatment.

## Introduction

Idiopathic inflammatory myopathy (IIM) is an autoimmune disease that affects the skeletal muscle system. It is often accompanied by elevated creatine kinase (CK), myositis-specific antibody positivity, and multisystemic involvement (1). The 239th ENMC Symposium, IIM was classified into four types: dermatomyositis, inclusion body myositis, immune-mediated necrotizing myopathy, and anti-synthetase antibody syndrome (2). In 2006, Pestronk et al. reported that a small proportion of patients with IIM present severe progressive atrophy of the arms and neck with mild involvement of the lower limbs and are represented by focal B-cell foci observed on histology (3). They suggested that Brachio-cervical inflammatory myopathy (BCIM) may be one of the independent subtypes of IIM, represented by focal B-cell foci observed on histology (3). Current studies on BCIM are limited and dominated by case reports. The University of Washington reported that the prevalence of BCIM syndrome was 0.3% in 4,130 muscle biopsy samples (3). It is important to note that BCIM is not representative of mild myositis symptoms. In 2015, Kooi et al. observed dilated cardiomyopathy as a potentially lethal complication of BCIM while following up on two female patients with BCIM (4). In 2018, academics from the University of Toronto described a patient with BCIM who also tested positive for scleroderma and lupus antibodies (5). This suggests that patients with BCIM may have a combination of fatal complications and immune system comorbidities. Early recognition and treatment are crucial for patient prognosis.

Flail arm syndrome (FAS) is a variant of amyotrophic lateral sclerosis (ALS) that can progress to classic ALS later in the course of the disease (6). The onset of FAS exhibits wide clinical variability. Previous studies have concluded that atrophy of the upper limb may be initiated at different sites: distal muscle groups (40%), both proximal and distal (36%), and purely proximal initial symptoms (24%) (7). However, no studies have reported misdiagnosis of FAS in patients with BCIM. We describe in details the diagnostic process of one case with BCIM. A summary of the differential diagnosis is also provided. Furthermore, the patient had rheumatoid arthritis, hypothyroidism, diabetes mellitus, and gout, which expanded the clinical spectrum and diagnostic experience of BCIM.

## Case description

The 55-year-old man had a 22-month history of bilateral upper limb and interosseous muscle atrophy, which had progressed to weakness in the neck muscles and fatigue when climbing stairs in the last three months. The patient also presented with atrophy of the bilateral upper limb and girdle muscles, particularly the trapezius, deltoids, supraspinatus, and pectorals. He also had a metacarpophalangeal joint deformity. We assessed the patient’s muscle strength using the Medical Research Council (MRC) grading system. The results showed that the biceps and triceps MRC grades were 3, the deltoid, trapezius, wrist extensors or flexors and iliopsoas MRC grade 4, and the facial muscles, gluteus maximus, hamstrings, tibialis anterior and tibialis posterior MRC grade 5. He had severely reduced muscle volume in his thoracic back and both upper extremities. No pain or numbness in the limbs was reported. In addition, there were no signs of upper motor neuron hyperactivity, including hyperreflexia, lingual tremor, palmar-mandibular reflex, Hoffman’s sign, and Babinski’s sign.

The patient has a medical history of rheumatoid arthritis for 10 years, gout for five years, and diabetes for one year. The laboratory examination revealed an elevated CK level ranging from 219 to 441U/L (normal range 200-400 U/L), an elevated rheumatoid factor (RF) at 195.98 IU/ml (normal range: 0-20 IU/ml), elevated anti-citrullinated antibodies at 134 IU/ml (normal range: 0-5 IU/ml), and serum anti-nuclear antibodies at a homogeneous 1:3200 titer. Serological tests for antibodies to myasthenia gravis are negative. Electromyography (EMG) revealed spontaneous fibrillation potentials and positive sharp waves in the muscles of the quadriceps, bicipital muscles, sternocleidomastoid, tibialis anterior, and rectus abdominis muscles, with no clues of RNS(repetitive nerve stimulation) abnormalities. An increased amplitude of motor unit potentials (MUPs) was observed in the right adductor hallucis longus muscle (**Table 1**). Magnetic resonance imaging (MRI) of the cervical and lumbar spine was performed to rule out any abnormalities in the spinal cord or intervertebral discs. Whole-genome sequencing and multiplex ligation-dependent probe amplification were performed to detect facioscapulohumeral dystrophy (FSHD), congenital myopathies, or other suspected genetic disorders. Computerized tomography (CT) scans of the head, chest, abdomen, and pelvis were conducted to detect any signs of metastatic diseases. Infection-related serological tests were conducted and the results were within normal limits.

**Table 1:** Changes in two Electroencephalogram results.

Muscles	EMG 1		MUPs		EMG 2		MUPs	
(Needle electromyography)	Fp	Psw	Dur	Amp	Fp	Psw	Dur	Amp
Left. little finger spreader muscle	**-**	**-**	10.9	1009	**+**	**-**	11.0	652
Right. abductor hallucis longus	**-**	**-**	10.4	2189 ↑	**-**	**+**	9.9	802
Left. quadriceps muscle group	**-**	**-**	12.8	1434	**++**	**++**	12.5	1319
Right. quadriceps muscle group	**++**	**++**	12.5	928	**++**	**++**	12.9	1076
Left. bicipital muscle	**++**	**++**	11.6	402	**++**	**++**	12.0	420
Right. bicipital muscle	**++**	**+**	11.9	505	**++**	**++**	9.9 ↓	598
Right. tibialis anterior muscle	**++**	**++**	14.0	600	**++**	**+++**	13.4	912
Left. tibialis anterior muscle	**++**	**++**	14.1	684	**++**	**+++**	13.6	1245
Left. sternocleidomastoid muscle	**-**	**+**	8.8	913	**-**	**+**	8.5	391
Left. gastrocnemius muscle	**++**	**++**	12.2	392	**+**	**++**	11.3	2317 ↑
Right. rectus abdominis muscle	**-**	**++**	/	/	**+**	**+**	/	/

The two EMG tests were conducted by the same experienced neurophysiologist at the same center. Two EMG tests were performed at six-month intervals. Fp, Fibrillation potential; Psw, positive sharp waves; Dur, Duration; Amp, Amplitude; EMG, Electromyography; MUPs, Motor Unit Potentials. -, absent; +, scattered; ++, moderate; +++, numerous; /, null; ↑, increased; ↓, decreased.

Based on the patient’s history of presenting with bilateral upper extremity weakness for over 12 months and absence of upper motor neuron damage, we suspected that the patient had early-onset amyotrophic lateral sclerosis with FAS. Consequently, we recommended that the patient take riluzole (50 mg, twice daily).

**Figure 1:**
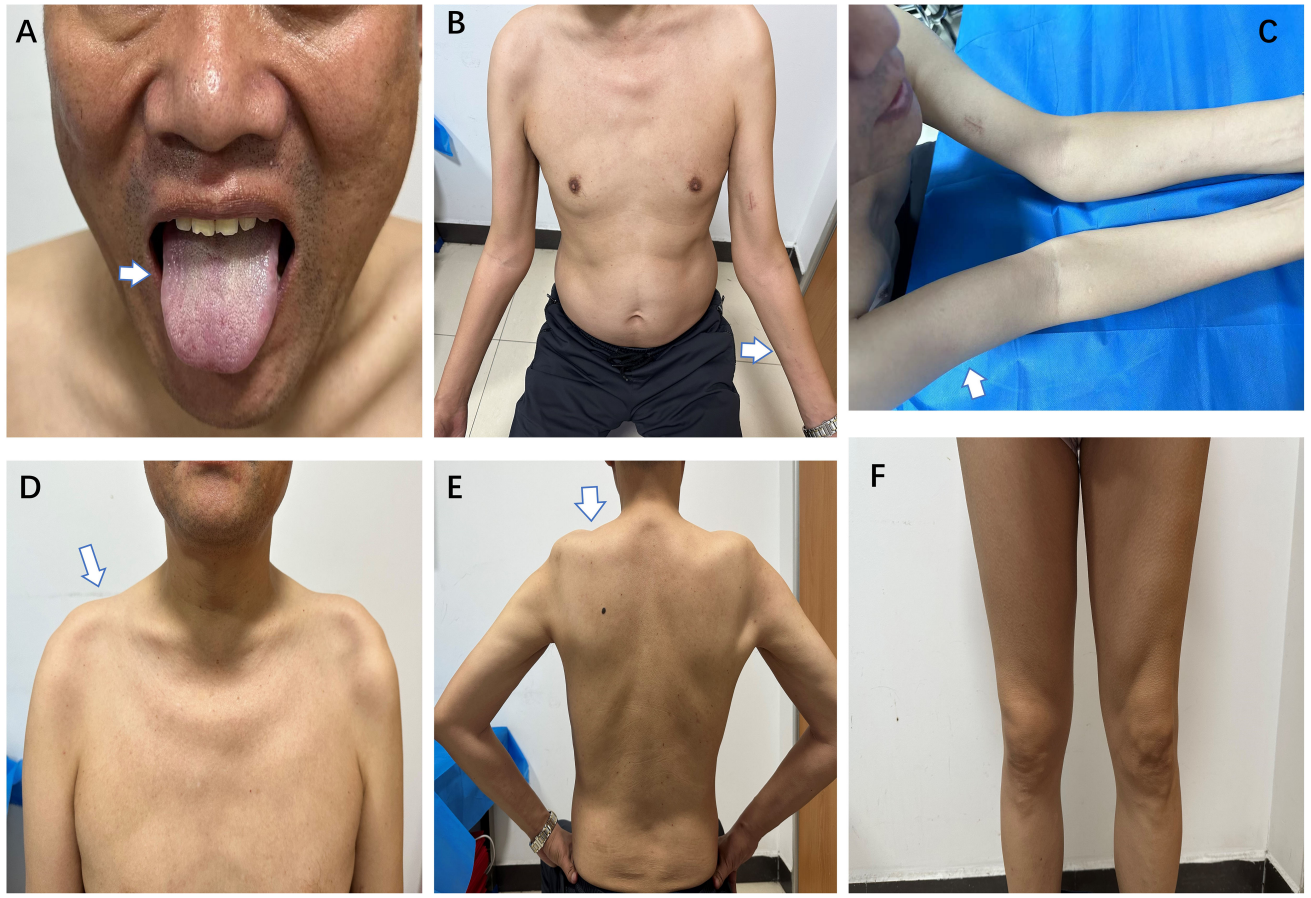
During the second admission, the patient had mild atrophy of the lingual muscles **(A)**, severe atrophy of both forearms **(B)**, upper arms **(C)**, and trapezius muscles **(D)** with concomitant shoulder collapse **(E)**, with preserved muscle volume in both lower extremities **(F)** (white arrows).

**Figure 2:**
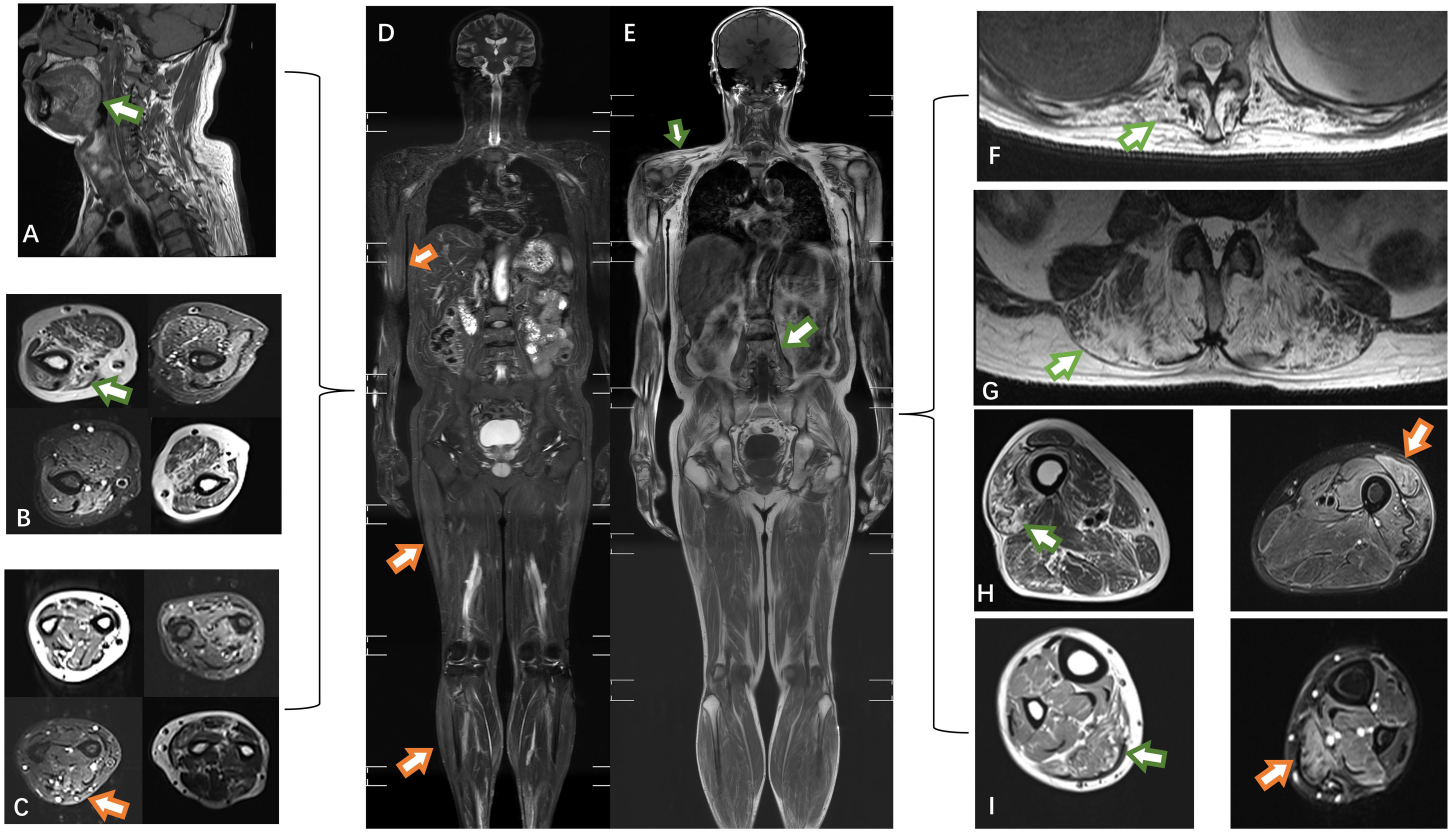
The muscles of the tongue showed mild lipoatrophy [**(A)**, green arrows]. Both the upper arm and forearm muscles exhibited bilateral asymmetric edema and lipoatrophy **(B, C)**. Edema was observed in the right biceps, lateral femoral, and gastrocnemius muscles (orange arrows) **(D)**. Atrophy was present in the trapezius and psoas muscles **(E)**. Severe fat replacement was noted in the dorsal muscles **(F, G)**. The volume of muscle in the lower extremities was preserved, with only slight involvement of the lateral group of thigh muscles compared to the posterior group of calf muscles **(H, I)**. T1-weighted imaging: green arrow; short-tau inversion recovery (STIR) MRI: orange arrow.

During the treatment period, the patient reported mild fluctuations in limb weakness. Six months later, the patient was readmitted to the hospital due for dysphagia. We reassessed the patient and found mild atrophy of the lingual muscles (**Figure 1A**), asymmetric involvement of the scapular winging (**Supplementary Figure 1**), worsening of amyotrophy (**Figures 1B–F**) and decreased strength of the quadriceps (MRC grade 4). The patient experienced difficulty swallowing 30 ml of room temperature water in a single sip, as evidenced by a water swallow test grade 3. A follow-up EMG conducted three months later showed a shortened MUP duration in the right biceps (9.9 ms, **Table 1**). Thus, a whole-body muscle MRI was performed, revealing diffuse edema in the patient’s upper arms and forearms bilaterally in short tau inversion recovery MRI. Mild edema was also observed in the patient’s right lateral femoral and gastrocnemius muscles (see **Figure 2**). The patient’s lingual muscles presented with mild fat replacement on T1-weighted imaging, while heavy adipose tissue infiltration was present in the upper limbs, back muscles, and psoas major. In contrast, the muscles of the lower limb exhibited less fat replacement, particularly in the lateral group of both thigh muscles and the posterior group of calf muscles. An MSA test was performed in peripheral blood for this condition, but the results were negative.

**Figure 3:**
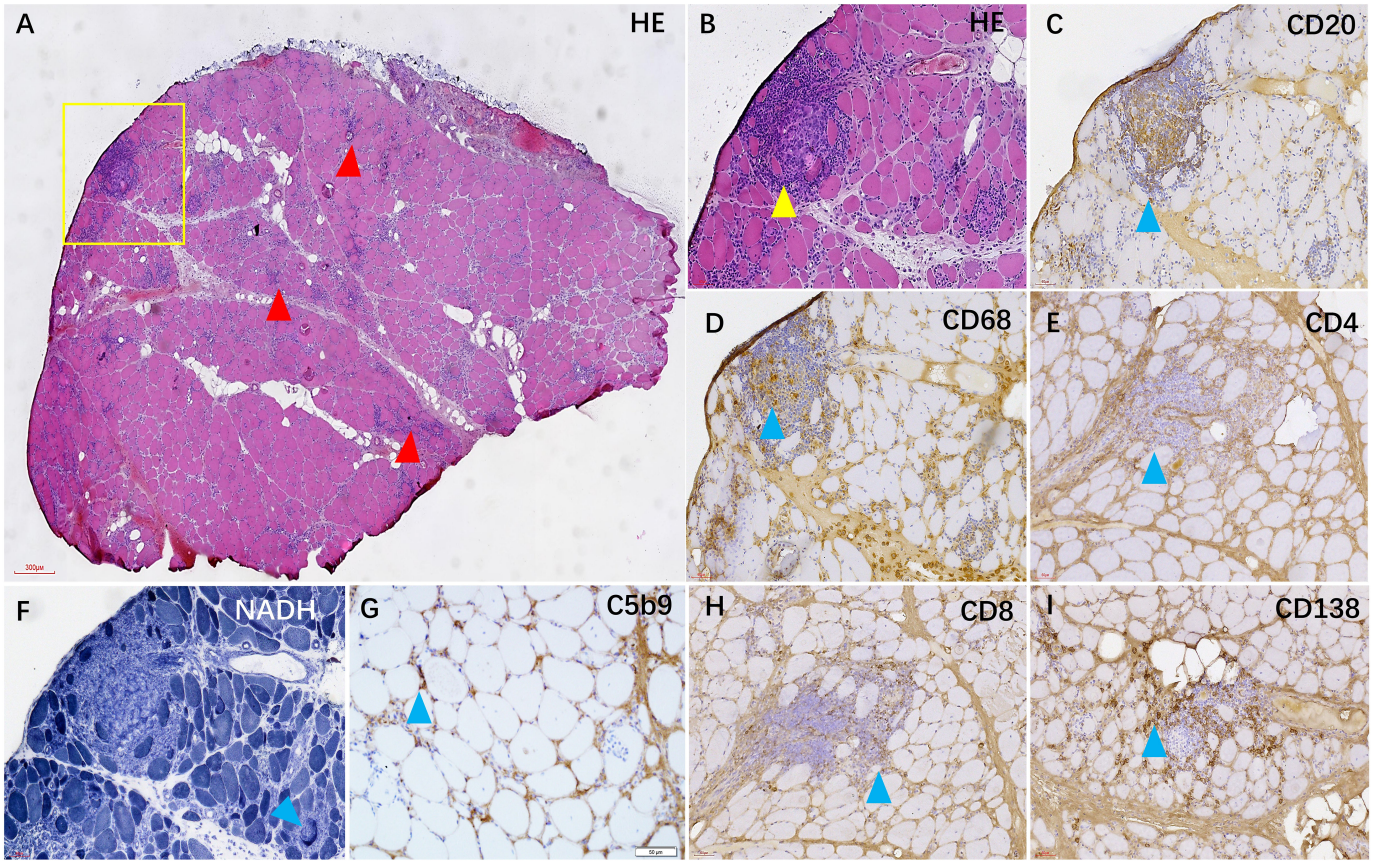
In the HE staining, numerous foci of inflammatory cells were observed in the muscle tissue [**(A, B)**, red and yellow arrows]. Immunohistochemical staining revealed that the foci were mainly composed of CD20+ B cells **(C)** and CD68+ macrophages **(D)**, accompanied by some CD138+ plasma cells **(I)** and a small number of CD4+ and CD8+ T cells **(E, H)**. Additionally, some myofibers exhibited heterogeneous NADH enzyme activity **(F)**, and C5b-9 was observed beneath the membrane of certain muscle fibers **(G)**.

A muscle biopsy was conducted on the patient’s biceps muscle to investigate the potential presence of myogenic damage. **Figure 3** illustrates the presence of numerous foci of inflammatory cells distributed within the muscle bundles, accompanied by a large number of necrotic and regenerating muscle fibers, moderate connective tissue hyperplasia, and mild fat cell replacement, as evidenced by hematoxylin and eosin (HE) staining. Immunohistochemical staining demonstrated an abundance of CD20+ B cells and CD68+ macrophages within the inflammatory aggregates, accompanied by moderate numbers of CD138+ plasma cells and scattered CD4+ and CD8+ T cells. Some myofibers exhibited uneven enzyme activity in NADH staining, and membrane attack complexes were deposited beneath the myofibers. In addition, MHC-I staining can be observed widely positive expression on myofibre membranes (**Supplementary Figure 2**). Metabolism myopathy was ruled out through oil red and glycogen staining.

The patient was ultimately diagnosed with BCIM and treated with oral prednisone (initially 60 mg/day, decrement) and methotrexate (5 mg/week). After four months, the patient reported mild improvement in swallowing function and low back strength. During this period, the patient also received treatment for rheumatoid arthritis, including 99m-Tc-methylene diphosphate (99Tc-MDP).

## Discussion

This article describes the misdiagnosis and diagnosis in a patient with BCIM. The patient initially presented with a FAS phenotype, in which the EMG indicated extensive denervation potentials with chronic nerve regeneration manifestations. During follow-up, only the right biceps brachii showed shortened duration of MUPs, while CK levels were consistently normal or lightly elevated, making the diagnosis of BCIM challenging.

Progressive bilateral upper extremity atrophy is the classic presentation of BCIM. It is important to differentiate the diagnosis from other conditions such as facioscapulohumeral dystrophy (FSHD), congenital myopathies, motor neuron disease, cervical spondylosis, and peripheral neuropathies. BCIM varies among individuals, but it can be summarized by the following five features: (1) The patient experiences weakness in both upper limbs, neck, and shoulders, with normal or mild involvement of the lower limbs, frequently accompanied by dysphagia. (2) Myositis-specific antibodies are absent (3); (3) The condition is often associated with other autoimmune diseases such as arthritis, systemic sclerosis, etc (8); (4) The muscle biopsy exhibits inflammatory cell foci dominated by CD20+ and CD68+ cells (5); (5) Other infections, heredity, or other pathogenic mechanisms have been excluded.

The patient’s first examination was characterized by lower motor syndrome damage that met the diagnostic criteria for FAS: progressive weakness of both upper extremities for more than 12 months, marked atrophy and subsidence of the shoulder due to the deltoid, supraspinatus, sternocleidomastoid, and teres minor, and absence of bulbar involvement and upper motor neuron symptoms (9, 10). Although the patient’s first electromyogram showed significant denervation potentials in the tibialis anterior and gastrocnemius muscles, denervation potentials in the lower extremities were not an exclusion criterion for FAS syndrome (10). Recently, it was observed that the IIM-associated “Dropped head or bent spine syndrome (DH/BS)” reported by Pijnenburg et al. is highly similar to the BCIM phenotype (11). Both syndromes involve delayed diagnosis, muscular atrophy of the upper limbs, dysphagia, and autoimmune abnormalities. Meanwhile, it was found that the prognosis and mortality rates were notably poorer in DH/BS patients compared to other IIM patients (11). However, we noticed that MSA antibodies reported by neurologists for BCIM should be negative, while MSA antibodies were present in a small subset of patients in the DH/BS cohort of rheumatologists represented by A et al. Unfortunately, due to the lack of biopsy data from a larger cohort, it is not possible to determine whether BCIM is part of DH/BS. Combined with our case, we observed neck muscle weakness in our patient, but not to the extent of head droop or spinal curvature. The characteristics of 14 patients with cervicobrachial polymyositis were summarized by Khadilkar et al. (12) In 50% of their cohort, muscle strength in the lower extremities was slightly affected, commonly in the psoas major, gluteus maximus, and quadriceps (12). Similarly, in last follow up, three out of six BCIM patients had lower limb involvement as reported by Lucchini et al. (13) This is consistent with our findings, suggesting that BCIM may not be confined to muscle involvement in the upper body. However, its unique differences in involvement and pathological manifestations make it difficult to be classified among the classical subtypes of IIM (2, 14, 15). More research is needed to investigate whether BCIM progresses to a generalized inflammatory myopathy at a later stage as the disease progresses.

It is worth noting that this individual had comorbid rheumatoid arthritis, which was a significant indication of BCIM. Previous reports have shown that patients with BCIM frequently exhibit a combination of Raynaud’s syndrome, Hashimoto’s thyroiditis, scleroderma (13), interstitial lung disease (16), and lupus serologically positive (5). Autoimmune-related markers are crucial in screening during the early stages. Additionally, the patient’s lumbar muscle involvement progressed from upper to lower extremities, indicating that BCIM may be a specific type of inflammatory myopathy with sequential involvement of sites. MRI characterization of the whole body allows for comparison of muscle involvement in the upper and lower extremities. Muscle biopsy is a valuable diagnostic tool for patients with bilateral upper extremity atrophy. In the patient’s second EMG findings, only the biceps brachii muscle showed duration of MUPs decreased. Widespread fibrillation potentials typically indicate neurogenic damage (17). However, muscle necrosis caused by myositis or myotonic dystrophy can also result in secondary loss of nerve manifestations (17). Therefore, we selected the biceps muscle for muscle biopsy and observed extensive foci of inflammatory cells. Given these considerations, repeating EMG testing in patients with lower motor neuron syndrome is worthwhile for both biopsy selection and differential diagnosis.

Currently, the classification and pathogenesis of BCIM remain unclear. Calvet et al. demonstrated that thrombospondin-1 (TSP-1) can potentially serve as a biomarker for BCIM, and its elevation promotes vascular disruption and myotube atrophy (16). Most patients with BCIM experience clinical and radiologic improvement with steroid and immunosuppressive therapy (13). CD20 and CD138 are enriched populations within inflammatory foci in the histology of BCIM. Targeting B-cell drugs, such as rituximab, could be an option for refractory BCIM, which binds to CD20 on the cell surface, utilizing antibody-dependent cytotoxicity, antibody-dependent cell phagocytosis, and direct apoptosis-inducing host effects to induce B-cell depletion (18). Furthermore, Telitacicept is a novel biological agent that binds and neutralizes two B lymphocyte stimulators and proliferation-inducing ligands. This inhibits the development and survival of both plasma cells and mature B cells (19). However, current second-line treatments, including anti-B-cell therapy, currently remain in the exploratory phase.

To summarize, it is essential to be aware that BCIM is one of the potential causes when patients present with the FAS phenotype. Due to the low prevalence of BCIM, we recommend that clinicians set a relatively low threshold for muscle biopsy in patients with atypical lower motor neuron syndrome and review electromyography at regular intervals. Despite the beneficial effect of immunotherapy on the patient in this case, continued follow-up is required to determine prognosis and potential relapse.

## Conclusion

Early FAS should be differentiated from BCIM. Serum immunologic markers, electromyography, whole-body muscle MRI, and muscle biopsy are ideal tools for diagnosing and evaluating treatment in patients with BCIM.

## Data availability statement

The raw data supporting the conclusions of this article will be made available by the authors, without undue reservation.

## Ethics statement

The studies involving humans were approved by Ethics Committee of First Affiliated Hospital of Jilin University. The studies were conducted in accordance with the local legislation and institutional requirements. The participants provided their written informed consent to participate in this study. Written informed consent was obtained from the individual(s) for the publication of any potentially identifiable images or data included in this article.

## Author contributions

HS: Conceptualization, Data curation, Investigation, Methodology, Supervision, Validation, Visualization, Writing – original draft, Writing – review & editing. X-JW: Data curation, Supervision, Writing – review & editing. YH: Data curation, Supervision, Writing – review & editing. Y-CW: Methodology, Writing – review & editing. Z-YW: Writing – review & editing. X-FY: Funding acquisition, Methodology, Resources, Supervision, Validation, Writing – review & editing.
