# Supplementary material for: Case report: A patient with brachio-cervical inflammatory myopathy was misdiagnosed as flail arm syndrome
Source: Front Immunol. 2024 Jul 3;15:1378130. doi: 10.3389/fimmu.2024.1378130 (PMC11251991; doi:10.3389/fimmu.2024.1378130)
Supplement: Supplementary file 1 [file DataSheet_1.docx]

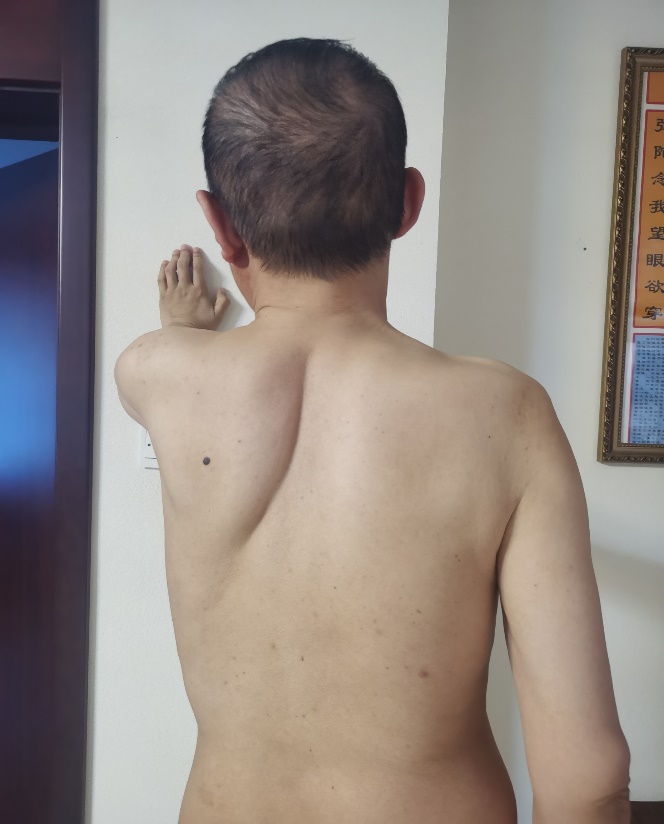

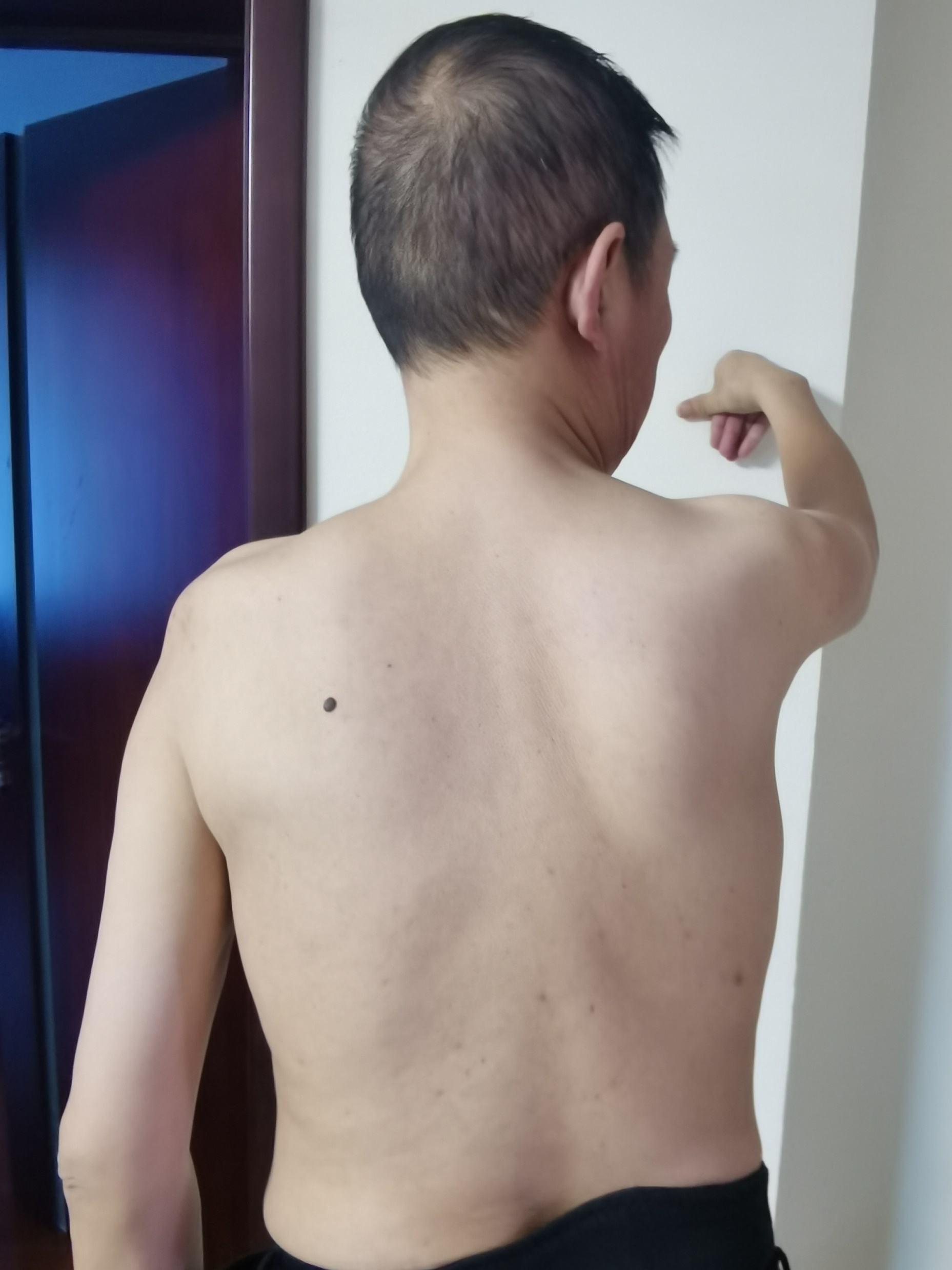


**A**

**B**

**Supplementary figure 1.** The patient had the scapular winging with asymmetric involvement (A and B).


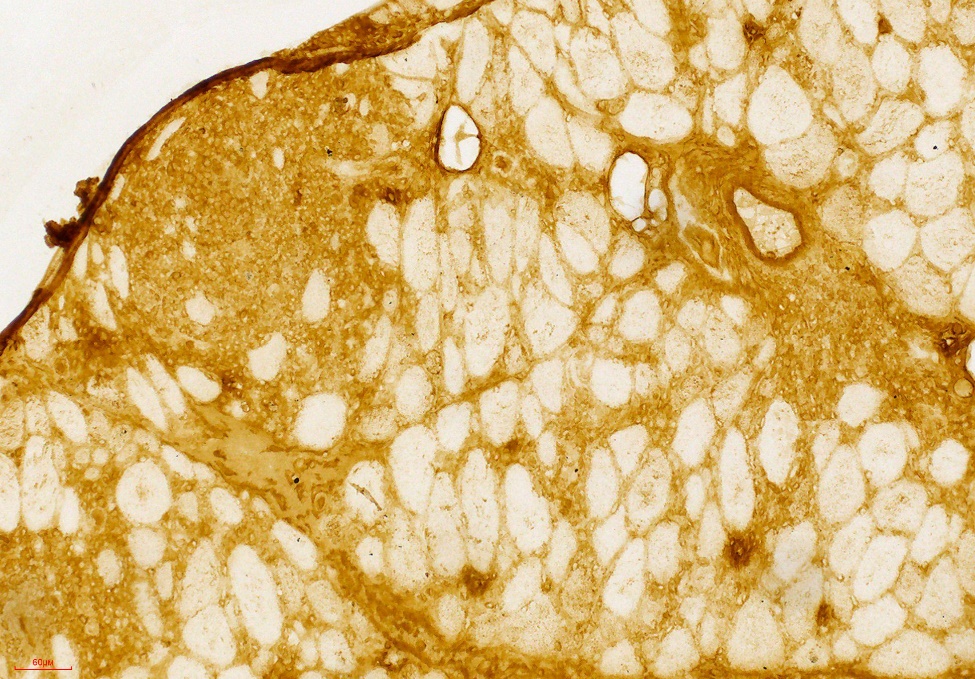


**Supplementary figure 2.** MHC-I staining revealed widespread positive expression on myofibre membranes.
